# Sub-Diffraction Limited Writing based on Laser Induced Periodic Surface Structures (LIPSS)

**DOI:** 10.1038/srep35035

**Published:** 2016-10-10

**Authors:** Xiaolong He, Anurup Datta, Woongsik Nam, Luis M. Traverso, Xianfan Xu

**Affiliations:** 1 School of Mechatronics Engineering, Harbin Institute of Technology, Harbin, Heilongjiang 150001, China; 2 School of Mechanical Engineering and Birck Nanotechnology Center, Purdue University, West Lafayette, IN 47907, USA

## Abstract

Controlled fabrication of single and multiple nanostructures far below the diffraction limit using a method based on laser induced periodic surface structure (LIPSS) is presented. In typical LIPSS, multiple lines with a certain spatial periodicity, but often not well-aligned, were produced. In this work, well-controlled and aligned nanowires and nanogrooves with widths as small as 40 nm and 60 nm with desired orientation and length are fabricated. Moreover, single nanowire and nanogroove were fabricated based on the same mechanism for forming multiple, periodic structures. Combining numerical modeling and AFM/SEM analyses, it was found these nanostructures were formed through the interference between the incident laser radiation and the surface plasmons, the mechanism for forming LIPSS on a dielectric surface using a high power femtosecond laser. We expect that our method, in particular, the fabrication of single nanowires and nanogrooves could be a promising alternative for fabrication of nanoscale devices due to its simplicity, flexibility, and versatility.

For the past decades, great progress has been achieved in ultrafast laser fabrication including femtosecond laser direct writing (FsLDW)^1^,^2^,^3^, making it a powerful tool to create two and three dimensional (2D and 3D) micrometer and sub-micrometer structures^4^,^5^ for photonic^6^,^7^,^8^, biomedical^9^,^10^, microfluidic^11^,^12^, and electronic applications^13^,^14^,^15^. In FsLDW, a femtosecond laser beam is tightly focused on the surface of or inside a material to remove or modify materials and form desired shapes^16^. By scanning the laser beam according to the designed routes, an entire 2D or 3D structure can be obtained^2^,^5^. While FsLDW is a convenient technique, the optical diffraction barrier limits the resolution and feature sizes to hundreds of nanometers^17^,^18^. There have been a great amount of research efforts to improve the fabrication resolution, such as zone-plate lithography^19^,^20^, two photon lithography^21^,^22^, and STED-DLW (stimulated emission depletion - direct laser writing)^23^,^24^,^25^. The resolution of zone-plate lithography and two photon lithography is usually limited to about 150 nm and 100 nm^26^,^22^, respectively, and STED-DLW involves more complex optical setups and photochemistry. On the other hand, with the discovery of laser induced periodic surface structures (LIPSS, also termed ripples), feature sizes below the diffraction limit were simply obtained^27^,^28^,^29^. LIPSS can be formed on surface of nearly all kinds of materials, including metals^30^,^31^, semiconductors^32^,^33^, and dielectrics^34^,^35^. These periodic structures typically are not well-aligned or sometimes random. These structures have found many applications such as super hydrophobic surface^36^, cell culture substrates^37^, and nonvolatile organic memory devices^38^. On the other hand, well-controlled, single structures will have many other applications.

In this work, we demonstrate a method to produce well-defined structures with nano-dimensions. The underlying mechanism is the formation of LIPSS on a dielectric surface, through the interference between the incident laser beam and the surface plasmons produced through a multi-photon absorption process. Electric-magnetic and thermal simulations are carried out to confirm the mechanism of forming the nanostructures. Unlike other LIPSS techniques where multiple, periodic lines and sometimes random structures are formed, this method has the capability to create nanowires or nanogrooves with desired quantity, length, and orientation. Moreover, single 60 nm-wide nanogroove and single 40 nm-wide nanowire are obtained based on the same LIPSS mechanism. This approach, in particular, the fabrication of single nanowire and nanogroove, could be a promising alternative for high-resolution nanofabrication such as fabricating nanoscale devices due to its simplicity, flexibility, and versatility.

## Results

**Figure 1:**
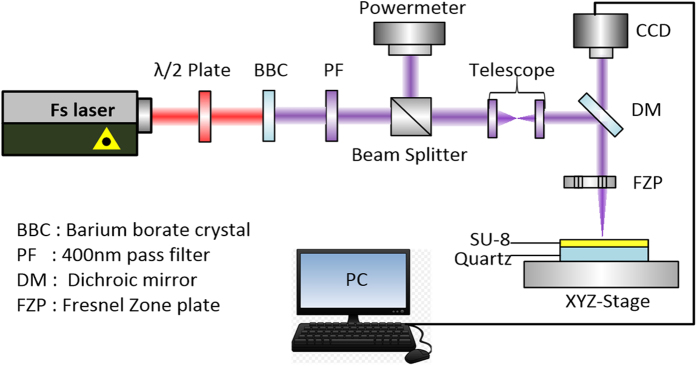
Schematic diagram of femtosecond laser-direct-writing.

The experimental set-up (Fig. 1 and Method) consists of a Ti: sapphire femtosecond laser, a beam delivery system, a home-made high numerical aperture Fresnel’s zone plates as focusing optics, CCD imaging system for monitor nanostructure fabrication process, and a piezo electric-stage and its control system. The sample is SU-8 (coated on quartz), a negative tone, chemically amplified resist, which has applications in various of fields due to its excellent planarization and processing properties as well as its mechanical and chemical stability^39^. Other aspects of the experiment are summarized in the Method and Supplementary Information.

### Effects of incident laser power

**Figure 2:**
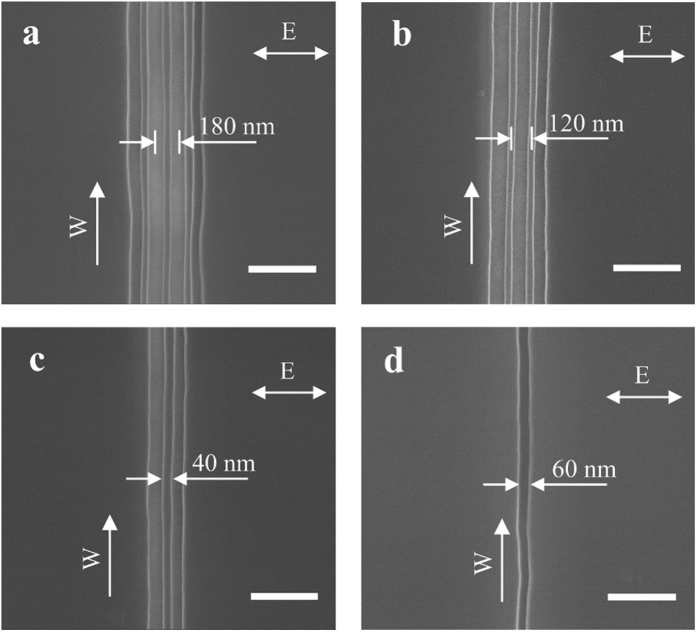
SEM images of the fabricated nanostructures. The incident laser fluences are (**a**) 0.03 J/cm^2^, (**b**) 0.02 J/cm^2^, (**c**) 0.015 J/cm^2^ and (**d**) 0.01 J/cm^2^, respectively. The arrow E indicates the laser polarization direction, arrow W indicates the writing direction, and all scale bars are 400 nm.

We first present the main experimental results: Fig. 2 shows the SEM images of the nanostructures produced by our method. The scanning speed of the piezoelectric stage is 0.5 μm/s. From the focused laser spot size and the laser pulse repetition rate, the number of pulses per laser spot is estimated to be 40 million. The laser exposure fluence varies from 0.01~0.03 J/cm^2^ (corresponding to peak intensity of 2.7 × 10^11^~8.1 × 10^11^ W/cm^2^), 0.01 J/cm^2^ for single nanogroove and 0.03 J/cm^2^ for quadruple nanogrooves. The details of the calculations are shown in the Supplementary Information S2. The laser polarization direction is set to be horizontal in the figure, and the writing direction (the direction for moving the sample on the piezoelectric state with respect to the stationary laser beam) is vertical. In Fig. 2a–c, by decreasing the laser exposure fluence, quadruple, triple, and double nanogrooves were obtained. In Fig. 2d, a single nanogroove with a width of 60 nm was obtained, which is less than 1/6^th^ of the laser wavelength of 395 nm. The distance between the centers of two adjacent nanogrooves is 180 nm, as shown in Fig. 2a. One can consider there is a nanowire between two nanogrooves, whose width is measured to be 40 nm which is about 1/10^th^ of the incident laser wavelength, and the distance between the centers of two neighboring nanowires is 120 nm, as shown in Fig. 2b. A single nanowire, also of 40 nm wide (surrounded by two nanogrooves), is shown in Fig. 2c. In Fig. 2a–d, the directions of all nanogrooves and nanowires are perpendicularly oriented to the laser polarization direction, and are in the same direction as the writing direction.

### Effects of laser polarization

**Figure 3:**
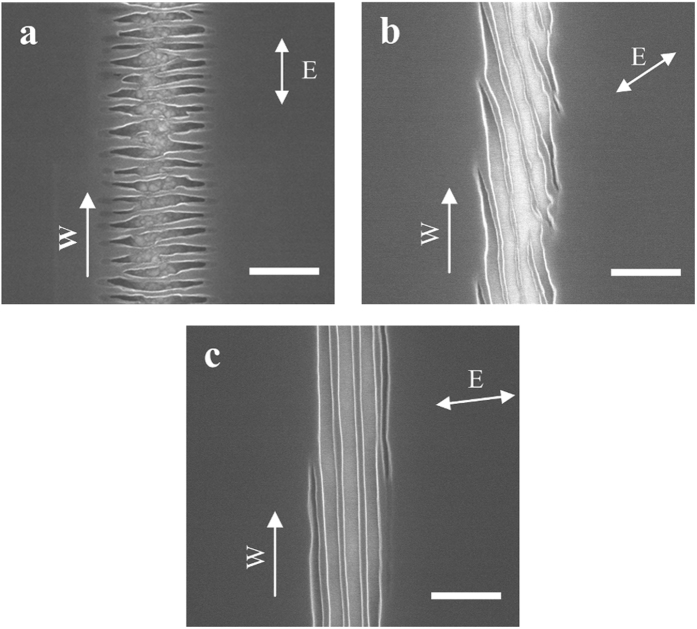
Effect of polarization direction on nanostructure fabrication. The incident laser fluence in (**a**,**b**,**c**) are the same, 0.03 J/cm^2^, and the polarization directions are (**a**) vertical, (**b**) 45 degrees and (**c**) 85 degrees from the vertical direction, respectively. The arrow E indicates the laser polarization direction, the arrow W indicates the writing direction and all scale bars are 400 nm.

The direction of laser polarization often plays a key role in determining the outcome of laser fabrication. To investigate the effect of the laser polarization direction on nanostructures’ shape and orientation, we change the polarization direction from horizontal to vertical, 45 degrees off vertical, and 85 degrees off vertical. The nanostructures obtained with these polarization directions are shown in Fig. 3. The structures in Fig. 3a have many short horizontal ripples. The short ripples in Fig. 3b are 45 degrees off the horizontal direction. In Fig. 3c, with polarization direction of the incident laser beam not completely perpendicular to the writing direction, nanogrooves and nanowires are formed but not continuous at the location shown in the figure, as the front of the leftmost groove moves outside of the laser focused area, and a new nanogroove starts to form from the right side. Combing Figs 2 and 3, we find that the direction of ripples (including nanogrooves and nanowires) are always perpendicularly oriented to the laser polarization direction.

### AFM images analysis

**Figure 4:**
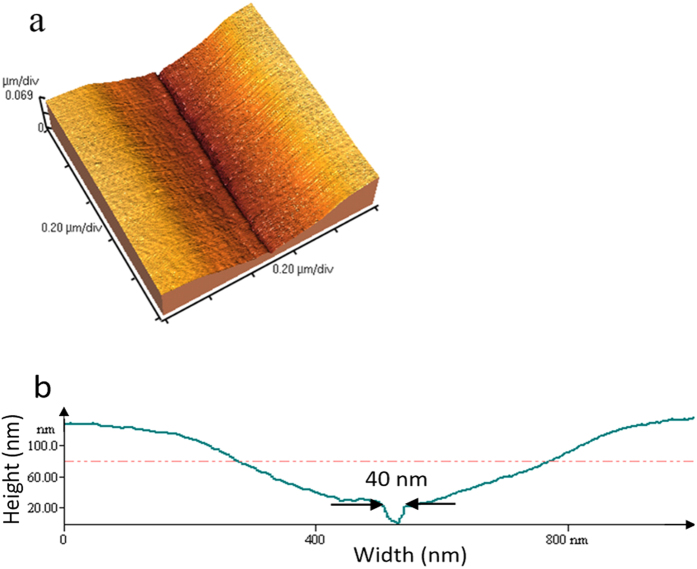
AFM image of nanostructures. The laser fluence is 0.01 J/cm^2^, (**b**) is the height profile of (**a**).

To further characterize these nanostructures, atomic force microscopy (AFM) images were taken to observe their height profiles. Figure 4a shows the AFM image of a single groove. It is seen that the nanogroove is within a gradually tapered, wider slot, which has a width of about 1 μm at the surface. Nanogrooves (and nanowires) are formed at the bottom of the slot, as shown in Fig. 4a,b. The width of nanogroove in AFM image is 40 nm, which is smaller than in SEM image, 60 nm. This is due to the finite size of the AFM tip, and the result of 40 nm is the convolution between the AFM tip and the actual topography. The tapered slot and the nano-grooves/wires are formed before developing the SU-8, therefore, these structures are formed through a thermal process: the laser power is sufficiently high to evaporate the SU-8 in and near the focal spot due to the Gaussian intensity distribution and thermal transport. On the other hand, the nanogrooves and nanowires are formed due to the interference effect between the incident laser beam and the surface plasmons (SPs), similar to the formation of LIPSS, which enhances the laser intensity locally. The single nanogroove is formed such that only the central interference fringe receives sufficient intensity for evaporating the SU-8 and forming a groove. After development, the profile of nanostructure has no change, since the SU-8 is a negative tone photoresist and any SU-8 exposed to laser irradiation (but not evaporated) is cross-linked and remains on the substrate.

## Discussion

We now discuss in details the mechanism for forming the nanogrooves and nanowires, but will first give a brief overview of the mechanisms of LIPSS formation. Upon irradiation of solids with linearly polarized (femtosecond) laser pulses, two distinct types of interference LIPSS patterns are usually observed, either parallel or perpendicular to the laser polarization direction^27^,^29^. The substrate material largely determines both the direction of ripples relative to the polarization direction as well as the periodicity (∧) of these ripples^29^. For strongly absorbing materials such as metals^30^,^31^ and semiconductors^32^,^33^, the orientation of ripples are generally perpendicular to the incident laser polarization direction, and ratio of the periodicity of these ripples to the laser wavelength (λ) is usually in the range of 0.4 < ∧/λ < 1. Hence, these ripples are called low-spatial-frequency LIPSS (LSFL). These LSFLs are believed to be generated by the interference between the incident laser radiation and surface plasmons caused by laser radiation^29^,^40^. LSFLs with orientation parallel to the polarization direction are usually observed in dielectrics^34^, and polymers materials^35^,^41^. These LSFLs form when the incident laser radiation interferes with scattered radiation from the substrate^42^. However, LSFLs with orientation perpendicular to the polarization direction can also be formed in dielectric materials, when high enough incident (femtosecond) laser pulses generate sufficient free electrons though multi-photon processes to cause the material acting metallically^43^,^44^. High spatial frequency LIPSS (HSFL, ∧/λ < 0.4) are prevailingly observed in dielectrics with orientations often perpendicular^45^, but sometimes parallel to the polarization direction^46^. The origin of the HSFL is still controversial, with possible restructuring of ripples acting as a feedback mechanism which decreases the ripple spacing as the number of laser pulses increases^29^,^46^. In our case, sufficient laser fluence (0.01~0.02 J/cm^2^) or peak intensity (2.7 × 10^11^~5.4 × 10^11^ W/cm^2^) is applied to the dielectric material SU-8 to make it behave metallically, and the nanostructures are formed due to the interference between the incident laser radiation and surface plasmons (see Numerical computations part below and Supplementary Information S2 for details). Therefore, the nanostructures are perpendicular to the laser polarization direction.

We further use numerical computations to illustrate the mechanism for forming the nanostructure discussed above. The computations consist of the following three parts: (1) the non-linear ultrafast pulse propagation in photoresist and the resulting change to the dielectric function, (2) the electromagnetic (EM) calculations of the interference effect, and (3) the temperature rise in the photoresist due to the absorption of the EM wave.

We modeled the non-linear propagation of the laser pulse inside SU-8 using a (2 + 1)-dimensional wave propagation equation^47^, and solved it simultaneously with the rate equation of the electron density. The electron density was then used to compute the dielectric function described by the Drude model to obtain the dielectric constant of SU-8. The details of the calculations are provided in the Supplementary Information S2. The incident laser fluence used for these calculations range from 0.01~0.02 J/cm^2^, corresponding to the formation of single and triple nanogrooves, respectively. For the laser power for forming a single nanogroove, the calculated free electron density is 1.65 × 10^21^ cm^−3^, which is very close to the critical electron density of SU-8 (1.67 × 10^21^ cm^−3^) obtaining from Drude model. Details of the Drude model calculation are provided in Supplementary Information S2. Using this electron density, the dielectric constant is calculated to be ε = −0.95 + 0.78i, which is close to the commonly accepted criterion for SPP excitation, Re(ε) < −1^40^. The discrepancy can be caused by uncertainties in the parameters used and inaccuracy in the numerical model. In addition, approximately 40 million pulses are applied at every point. That many pulses will likely generate defects and increase absorption. As a result, more carriers can be generated, leading to a more metallic behavior of the surface. This in turn will assist the formation of the interference between surface plasmons and incident laser radiation.

**Figure 5:**
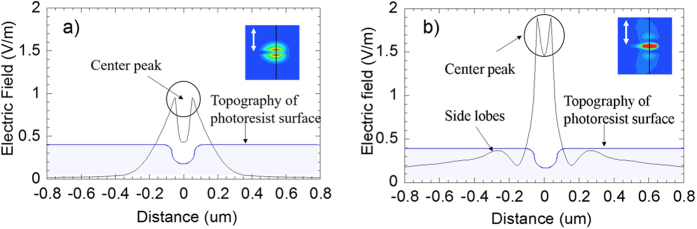
Computational results of electrical field (|Ex|^2^) distributions. (**a**) 0.01 J/cm^2^ incident laser fluence, (**b**) 0.02 J/cm^2^ laser fluence. The white arrows in the inset images indicate the laser polarization direction.

Using the calculated dielectric constant, we then computed the electromagnetic field distribution using the frequency-domain finite-element method (FEM)^48^ (see Supplementary information S3 for details). Figure 5 shows the calculated electric field intensity profile along a cross section line of the nanogrooves, Fig. 5a for 0.01 J/cm^2^ incident laser fluence and Fig. 5b for 0.02 J/cm^2^. The inset figures depict the electric field polarization direction denoted by the white arrow and the electric field intensity distribution at the surface of the photoresist, and the cross section is taken along the black line shown in the inset figures. The surface topography profile is also included in the figures along with the electric field intensity profile. In Fig. 5a, the center peak has two high tips, caused by light scattered from the groove edges. For the case shown in Fig. 5b, there are a center peak and two side lobes caused by the interference effect. The distance between the center and each side lobe is about 180 nm, consistent with the experimental observation. The fringes formed by the interference are perpendicular to the laser polarization direction (inset in Fig. 5a,b), which is also consistent with the experiment results and the theoretical explanation.

**Figure 6:**
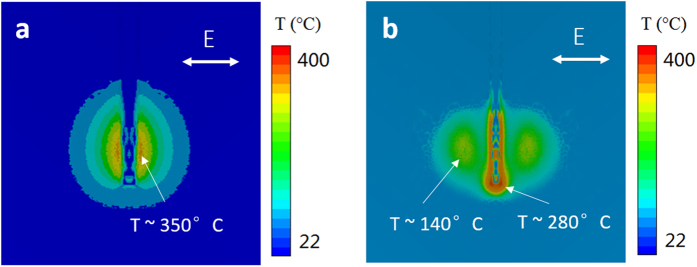
Temperature distribution of the surface at the end of a single laser pulse for (**a**) laser fluence of 0.01 J/cm^2^ and (**b**) laser fluence of 0.02 J/cm^2^.

The temperature rise in the SU-8 was estimated using a finite element analysis, taking the result of laser energy absorption profile from the electromagnetic calculation as the input (see Supplementary Information S4 for details). With the incident laser fluence of 0.01 J/cm^2^, the temperature rise at the center peak was found to be about 350 °C (Fig. 6a), exceeds the boiled point of SU-8 (130 °C)^49^. With the increase in the laser fluence, the interference effect between the incident and surface plasmons become more prominent, and when the laser fluence increases to 0.02 J/cm^2^, the temperature rises at two side lobes reach 140 °C, also exceeds the boiling point (Fig. 6b). Figure 6b also shows that the region where the temperature is the highest is located at the tip of the groove, about 280 °C. This high temperature region at the groove tip, together with the two side lobes lead to the forming of triple nanogrooves.

**Figure 7:**
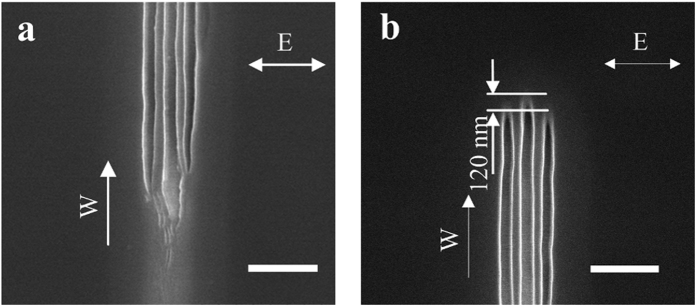
SEM images of nanostructure formation process. (**a**) Forming at the beginning of the nanostructure which produces some random ripples until the stable interference is generated. (**b**) Well aligned nanogrooves at the termination end of the nanostructure, the side nanogrooves are behind of the center nanogroove. The arrow E indicates the laser polarization direction, arrow W indicates the writing direction, and all scale bars are 400 nm.

The explanation given above is further supported by noting the beginning and the end of the nanostructures. At the beginning, there are no surface plasmons and no interference before any nanostructure is formed (surface plasmons cannot be formed on a perfectly smooth surface). Without interference, the initial laser irradiation produces somewhat random ripples as shown in Fig. 7a. Interference, and thus continuous nanogrooves and nanowires are only formed after an initial period of laser irradiation, also shown in Fig. 7a. On the other hand, when the writing of nanostructures is terminated by blocking the laser beam, the end of the nanostrcutures is a representation of the laser power distribution at the instant when the laser irradiation is terminated. Figure 7b shows the end of the triple nanogrooves, which are well-aligned, with no randomness as in Fig. 7a. However, the center groove is leading the two side grooves, which is about 125 nm further along the scanning direction. This in fact is consistent with the result of the EM and thermal simulations, where the highest intensities of the side lobes are located about 120 nm behind the tip of the groove where the field intensity and the temperature are the highest (Figs 5b and 6b). Therefore, the images showing the beginning and the end of nanowire formation are all consistent with the mechanism of nanowire formation.

In conclusion, we developed a controllable method for fabricating nanostructures far below the diffraction limit. We utilized the interference between the incident laser radiation and the surface plasmons formed from multi-photon absorption to produce these nanostructures with tens of nanometers feature sizes. Multiple nanogrooves with 180nm period and nanowires with 120 nm period were produced. Single nanostructures, 60-nm wide nanogrooves and 40 nm-wide nanowires were obtained based on the same mechanism. Numerical calculations including ultrafast pulse propagation, electrical field distribution, and temperature rise agreed with the phenomena observed in the experiments. Our approach has the capability to create single and multiple nanostructures far below the diffraction limit with specific length and orientation, which can serve as a promising nano-fabrication method for manufacturing nanostructured components and devices.

## Methods

The schematic diagram of the experimental method is shown in Fig. 1, which consists of a femtosecond laser, a beam delivery system, home-made high numerical aperture Fresnel’s zone plates as focusing optics, CCD imaging system for monitor nanostructure fabrication process, and a piezo electric-stage and its control system. A Coherent Mira Ti: sapphire femtosecond laser which has a center wavelength of 790 nm, a repetition rate of 80 MHz and a pulse duration of 38 fs was used as the light source. A half wave plate was used to control the power for the experiment and control the laser polarization direction. The laser beam was frequency doubled by a BBO crystal to a wavelength of 395 nm and is then focused onto SU-8, which is the material we used for forming nanostructures. The 395 nm beam then goes through a telescope to reduce its diameter and increase the intensity on a Fresnel’s zone plate, which focuses the laser beam onto the substrate. The zone plate has a focal length of 50 μm, a numerical aperture of 0.95, producing a diffraction limited spot size of about 250 nm (see Supplementary Information S1 for details). The substrate used for forming nanowire is 1 mm-thick quartz, and it is fixed on a Mad City Labs piezoelectric stage. The piezoelectric stage scans in both the X and Y directions, and has a resolution of 0.1 nm and a typical noise of about 10 nm during operation. A CCD camera was used to verify the position of the laser on the zone plates and monitor nanostructure fabrication.

The sample SU-8 2000.2 was spin-coated on the quartz substrate to a thickness of about 200 nm. The substrate was first cleaned with acetone and isopropanol to remove debris and dust. SU-8 was then dropped cast onto the substrate, spin-coated with 3000 rpm for 1 min, then soft-baked at 95 °C for 1 min. After exposure, the sample was immersed in SU-8 developer for 1 min and rinsed with isopropanol for 30 s. Nanostructures in exposed area were observed by a Hitachi S-4800 Field Emission Scanning Electron Microscopy (FESEM) and a Thermo Microscopes Autoprobe CP Atomic Force Microscope (AFM), and the widths were determined using SEM images in combination with an imaging software tool.

## Additional Information

**How to cite this article**: He, X. *et al*. Sub-Diffraction Limited Writing based on Laser Induced Periodic Surface Structures (LIPSS). *Sci. Rep*. **6**, 35035; doi: 10.1038/srep35035 (2016).

## Supplementary Material

Supplementary Information

## References

[b1] Keller U. Recent developments in compact ultrafast lasers. Nature 424, 831–838 (2003). 12917697 10.1038/nature01938

[b2] Anscombe N. Direct laser writing. Nat. photonics 4, 22–23 (2010).

[b3] Sugioka K. & Cheng Y. Ultrafast lasers—reliable tools for advanced materials processing. Light Sci. Appl. 3, e149 (2014).

[b4] Vorobyev A. Y. & Guo C. L. Direct femtosecond laser surface nano / microstructuring and its applications. Laser Photonics Rev. 7, 385–407 (2013).

[b5] Zhang Y. L., Chen Q. D., Xia H. & Sun H. B. Designable 3D nanofabrication by femtosecond laser direct writing. Nano Today 5, 435–448 (2010).

[b6] Gattass R. R. & Mazur E. Femtosecond laser micromachining in transparent materials. Nat. Photonics 2, 219–225 (2008).

[b7] Zeng H. . High-Resolution 3D Direct Laser Writing for Liquid-Crystalline Elastomer Microstructures. Adv. Mater. 26, 2319–2322 (2014). 24421068 10.1002/adma.201305008

[b8] Stone A. . Direct laser-writing of ferroelectric single-crystal waveguide architectures in glass for 3D integrated optics. Sci. Rep. 5, 10391 (2015). 25988599 10.1038/srep10391 PMC4437375

[b9] Melissinaki V. . Direct laser writing of 3D scaffolds for neural tissue engineering applications. Biofabrication 3, 045005 (2011). 21931197 10.1088/1758-5082/3/4/045005

[b10] Sugioka K., Hanada Y. & Midorikawa K. Three-dimensional femtosecond laser micromachining of photosensitive glass for biomicrochips. Laser Photon. Rev. 3, 386–400 (2010).

[b11] McDonald J. P., Mistry V. R., Ray K. E. & Yalisove S. M. Femtosecond pulsed laser direct write production of nano- and microfluidic channels. Appl Phys Lett 88, 183113 (2006).

[b12] Sugioka K. . Femtosecond laser 3D micromachining: a powerful tool for the fabrication of microfluidic, optofluidic, and electrofluidic devices based on glass. Lab Chip 14, 3447–3458 (2014). 25012238 10.1039/c4lc00548a

[b13] Deubel M. . Direct laser writing of three-dimensional photonic-crystal templates for telecommunications. Nat. Mater. 3, 444–447 (2004). 15195083 10.1038/nmat1155

[b14] Gao W. . Direct laser writing of micro-supercapacitors on hydrated graphite oxide films. Nat. Nanotechnol. 6, 496–500 (2011). 21804554 10.1038/nnano.2011.110

[b15] El-Kady M. F. & Kane’ r R. B. Direct laser writing of graphene electronics. ACS Nano 8, 8725–8729 (2014). 25215512 10.1021/nn504946k

[b16] Arnold C. B. & Piqué A. Laser direct-write processing. MRS Bull. 32, 9–15 (2007).

[b17] Ushiba S., Shoji S., Masui K., Kono J. & Kawata S. Direct laser writing of 3D architectures of aligned carbon nanotubes. Adv. Mater. 26, 5653–5657 (2014). 24944112 10.1002/adma.201400783

[b18] Sakellari I. . Diffusion-assisted high-resolution direct remtosecond laser writing. ACS Nano, 6, 2302–2311 (2012). 22324511 10.1021/nn204454c

[b19] Chao D., Patel A., Barwicz T., Smith H. I. & Menon R. Immersion zone-plate-array lithography. J. Vac. Sci. Technol. B 23, 2657–2661 (2005).

[b20] Smith H. I. . Zone-plate-array lithography: a low-cost complement or competitor to scanning-electron-beam lithography. Microelectron. Eng. 83, 956–961 (2006).

[b21] Kawata S., Sun H. B., Tanaka T. & Takada K. Finer features for functional microdevices, Nature 412, 697–698 (2001). 11507627 10.1038/35089130

[b22] Farsari M. & Chichkov B. N. Materials processing: two-photon fabrication, Nat. Photon. 3, 450–452 (2009).

[b23] Li L. J., Gattass R. R., Gershgoren E., Hwang H. & Fourkas J. T. Achieving λ/20 resolution by one-color initiation and deactivation of polymerization. Science 324, 910–913 (2009). 19359543 10.1126/science.1168996

[b24] Gan Z. S., Cao Y. Y., Evans R. A. & Gu M. Three-dimensional deep sub-diffraction optical beam lithography with 9 nm feature size. Nat. Commun. 4, 2061 (2013). 23784312 10.1038/ncomms3061

[b25] Buchegger B. . Stimulated Emission Depletion Lithography with Mercapto-Functional Polymers. ACS Nano 10, 1954–1959 (2016). 26816204 10.1021/acsnano.5b05863 PMC4768287

[b26] Gil D., Menon R. & Smith H. I. The Case for Diffractive Optics in Maskless Lithography. J. Vac. Sci. Technol. B 21, 2810–2814 (2003).

[b27] Sipe J. E., Young J. F., Preston J. S. & van Driel H. M. Laser-induced periodic surface structure. I. Theory, Phys. Rev. B 27, 1141–1154 (1983).

[b28] Young J. F., Preston J. S., van Driel H. M. & Sipe J. E. Laser-induced periodic surface structure. II. Experiments on Ge, Si, Al, and brass, Phys. Rev. B 27, 1155–1172 (1983).

[b29] Bonse J., Krüger J., Höhm S. & Rosenfeld A. Femtosecond laser-induced periodic surface structures. J. Laser Appl. 24, 042006 (2012).

[b30] Öktem B. . Nonlinear laser lithography for indefinitely large-area nanostructuring with femtosecond pulses. Nat. Photonics 7, 897–901 (2013).

[b31] Zhang Y. Q. . Plasmonic hybridization induced trapping and manipulation of a single Au nanowire on a metallic surface. Nano Lett. 14, 6430–6436 (2014). 25302534 10.1021/nl502975k

[b32] Derrien T. J. Y. . Rippled area formed by surface plasmon polaritons upon femtosecond laser double-pulse irradiation of silicon. Opt. express 23, 29643–29655 (2013). 10.1364/OE.21.029643 24514516

[b33] Kuladeep R., Sahoo C. & Rao D. N. Direct writing of continuous and discontinuous sub-wavelength periodic surface structures on single-crystalline silicon using femtosecond laser. Appl. Phys. Lett. 104, 222103 (2014).

[b34] Liu Y. . Ciliary White Light: Optical Aspect of Ultrashort Laser Ablation on Transparent Dielectrics. Phys. Rev. Lett. 110, 097601 (2013). 23496745 10.1103/PhysRevLett.110.097601

[b35] Rebollar E., Castillejo M. & Ezquerra T. A. Laser induced periodic surface structures on polymer films: From fundamentals to applications. Eur. Polym. J. 73, 162–174 (2015).

[b36] Long J. Y. . Superhydrophobic and colorful copper surfaces fabricated by picosecond laser induced periodic nanostructures. Appl. Surf. Sci. 311, 461–467 (2014).

[b37] Rebollar E. . Physicochemical modifications accompanying UV laser induced surface structures on poly (ethylene terephthalate) and their effect on adhesion of mesenchymal cells, Phys. Chem. Chem. Phys. 16, 17551–17559 (2014). 25025655 10.1039/c4cp02434f

[b38] Martínez-Tong D. E. . Laser fabrication of polymer ferroelectric nanostructures for nonvolatile organic memory devices. ACS Appl. Mater. Interfaces 7, 19611–19618 (2015). 26280158 10.1021/acsami.5b05213

[b39] Campo A. D. & Greiner C. SU-8: a photoresist for high-aspect-ratio and 3D submicron lithography. J. Micromech. Microeng. 2007 ; 17, 81–95 (2007).

[b40] Huang M., Zhao F. L., Cheng Y., Xu N. S. & Xu Z. Z. Origin of laser-induced near-subwavelength ripples: interference between surface plasmons and incident laser. ACS Nano 3, 4062–4070 (2009). 20025303 10.1021/nn900654v

[b41] Zhai T. R., Zhang X. P., Pang Z. G. & Dou F. Direct writing of polymer lasers using interference ablation. Adv. Mater. 23, 1860–1864 (2011). 21374741 10.1002/adma.201100250

[b42] Mitchell J. I., Zhou N., Nam W., Traverso L. M. & Xu X. Sub-diffraction laser synthesis of silicon nanowires. Sci. Rep. 4, 3908 (2014). 24469704 10.1038/srep03908 PMC3904146

[b43] Liao Y. . Femtosecond laser nanostructuring in porous glass with sub-50 nm feature sizes. Opt. lett. 38, 187–189 (2013). 23454957 10.1364/OL.38.000187

[b44] Liu J. K. . Direct writing of 150 nm gratings and squares on ZnO crystal in water by using 800 nm femtosecond laser. Opt. express 22, 32361–32370 (2014). 25607200 10.1364/OE.22.032361

[b45] Rohloff M. . Formation of laser-induced periodic surface structures on fused silica upon multiple cross-polarized double-femtosecond-laser pulse irradiation sequences. J. Appl. Phys. 110, 014910 (2011).

[b46] Jia T. Q. . Formation of nanogratings on the surface of a ZnSe crystal irradiated by femtosecond laser pulses. Phys. Rev. B 2005 ; 72, 125429 (2005).

[b47] Wu A. Q., Chowdhury I. H. & Xu X. Femtosecond laser absorption in fused silica: Numerical and experimental investigation. Phys. Rev. B 72, 085128 (2005).

[b48] Austin D. R. . Laser induced periodic surface structure formation in germanium by strong field mid IR laser solid interaction at oblique incidence. Opt. express 23, 19522–19534 (2015). 26367610 10.1364/OE.23.019522

[b49] Material Safety Data Sheet. MICRO CHEM (19/04/2012) [PDF on Internet]. Available from: https://louisville.edu/micronano/files/documents/safety-data-sheets-sds/SU82000.pdf. Date of access: 02/09/2016.

