# Supplementary material for: Sub-Diffraction Limited Writing based on Laser Induced Periodic Surface Structures (LIPSS)
Source: Sci Rep. 2016 Oct 10;6:35035. doi: 10.1038/srep35035 (PMC5056352; doi:10.1038/srep35035)
Supplement: Supplementary Information [file srep35035-s1.pdf]

Supplementary information for:

Sub-Diffraction Limited Writing based on Laser Induced Periodic Surface Structures (LIPSS)

Xiaolong He[1,2], Anurup Datta[2], Woongsik Nam[2], Luis M. Traverso[2] and Xianfan Xu[2,*]

[1]School of Mechatronics Engineering, Harbin Institute of Technology, Harbin, Heilongjiang 150001, China

[2]School of Mechanical Engineering and Birck Nanotechnology Center, Purdue University, West Lafayette, IN 47907, USA

Email address of authors: he274@purdue.edu, datta0@purdue.edu, namw@purdue.edu, ltravers@purdue.edu and xxu@purdue.edu

Correspondence: Professor X Xu, 585 Purdue Mall, School of Mechanical Engineering, Purdue University, West Lafayette, IN 47907, USA

E-mail: xxu@ecn.purdue.edu

Phone: (765) 494-5639

Fax: (765) 494-0539

1 Fresnel's zone plate

Fresnel's zone plate is an optical element for focus light through diffraction instead of refraction or reflection.[1] A zone plate consists of a set of radially symmetric rings, which are spaced so that the diffracted light constructively interferes at the desired focus. Microfabrication technique such as electron beam lithography is one of the best methods for fabricating microscale zone plates, which is capable of making features as small as 10 nm and registration errors to 1 nm.[2] In this work, we use electron beam lithography to fabricate zone plates. The total dimension of the zone plate is 300 μm in diameter, designed to have a focal length of 50 μm and a numerical aperture of 0.95. SEM image of a zone plate with 30 degree tilted and a zoom in image are shown in Figure S1.

The diffraction efficiency of our zone plate was calculated as the ratio of the output power in the focal spot and the total incident power on the zone plate. To get the output power at the focal spot, we evaluated the diffracted field distribution at the focal plane using the method by Cao and Jahns,[3] and integrated the diffracted power over the focused spot. The efficiency was 15.8 percent. This efficiency was then used to compute the laser fluence at the focus spot.

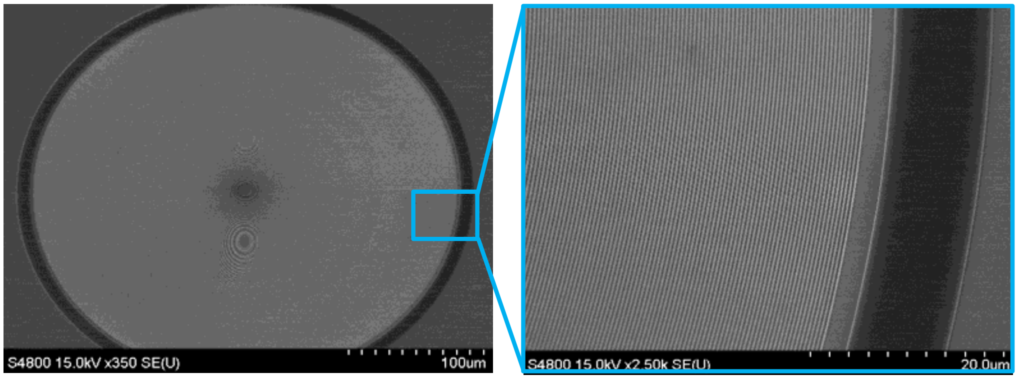

Figure S1. Fresnel zone plate fabricated by electron beam lithography.

2 Calculations of non-linear ultrafast pulse propagation in SU-8

The laser power is measured before the zone plate to be 2.7 mW for single nanogrooves formation and 5.4 mW for triple nanogrooves formation. The zone plate efficiency is 15.8%. With a focal spot diameter from zone plate of 250 nm, repetition rate of the laser pulses (80 MHz), pulse duration of 38fs, and scan speed of 0.5 um/s, we can obtain the number of pulses per laser spot diameter, which is 40M /spot diameter. Then, it was found the single pulse energy at the focal point is about 0.005 nJ, the laser fluence is about 0.01 J/cm^2 and a peak intensity of 2.7×10^{11} W/cm^2. For triple nanogrooves formation, the single pulse energy is about 0.01 nJ, the laser fluence is about 0.02 J/cm^2 and a peak intensity of 5.4×10^{11} W/cm^2.

Simulations of single pulse propagation in SU-8 were performed using a (2+1)-dimensional wave propagation equation,[4] considering the effect of laser beam diffraction, group velocity dispersion, self-focusing, defocusing, and nonlinear photoionization, which was solved simultaneously with the rate equation of the electron density. Some key parameters of SU-8 used for the calculation here are the initial refractive index 1.65,[5] and a band gap of 3.4eV,[6] and the effective mass of electron 0.12 m_e.[7] The electron density obtained then is used to compute the dielectric function of SU-8 following procedures in Ref. 4. With incident laser fluence for fabricating single nanogroove 0.01 J/cm^2 , the maximum electron density is found to be 1.65×10^{21} cm^{-3}. Then, the dielectric constant is calculated to be $\varepsilon = -0.95 + 0.78i$, from Drude model using the method described in Ref. 4. With a higher laser fluence of 0.02 J/cm^2 , the electron density reaches 6.49×10^{21} cm^{-3} and the dielectric constant $\varepsilon = -11.71 + 3.07i$. The distribution of electron density and absorption coefficient are shown in Figure S2.

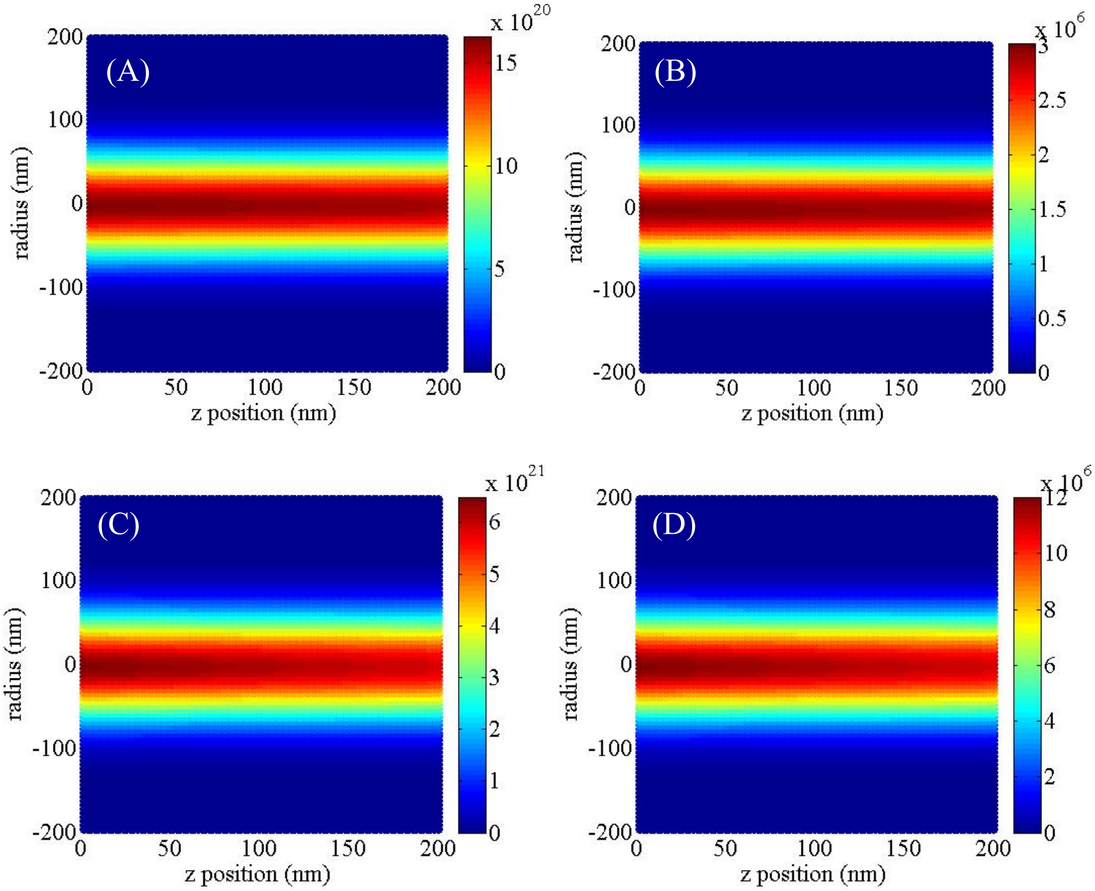

Figure S2: Light propagation in SU-8 with 400 nm wavelength and 250 nm light spot diameter. A) Electron density and B) absorption coefficient for a laser fluence of 0.01 J/cm^2, C) electron density and D) absorption coefficient for a laser fluence of 0.02 J/cm^2.

3 Electromagnetic calculations of the interference between surface plasmon and the incident laser beam

Using the modified dielectric constant of the photoresist SU-8, full wave electromagnetic simulation was carried out using a finite-element method (FEM) based commercial software, ANSYS HFSS version 15.0. The model consisted of a 200 nm thickness SU-8 photoresist layer on a quartz substrate. Nanogrooves were made on the SU-8 surface depending on the laser fluence, the dimensions of which were taken from the SEM images. In the following simulation, the incident Gaussian beam of diameter 250 nm, was used and its polarization direction was set

perpendicular to the axis of the nanogroove. Based on the symmetry of the model, only half of it was numerically solved to save computational resources. Radiation boundary conditions were assumed at the outer surface of the model while at the symmetry plane, boundary condition corresponding to zero tangential electric field was considered. Figure S3A and Figure S3B shows the intensity distribution at the surface of the photoresist corresponding to the laser fluence of 0.01 J/cm^2 and 0.02 J/cm^2, respectively. In Fig. 3Sa, the two hot spots are at the edge of the groove due to scattering. The interference effect between the incident light and the surface plasmons becomes prominent giving rise to distinct side lobes as seen in Figure S3B.

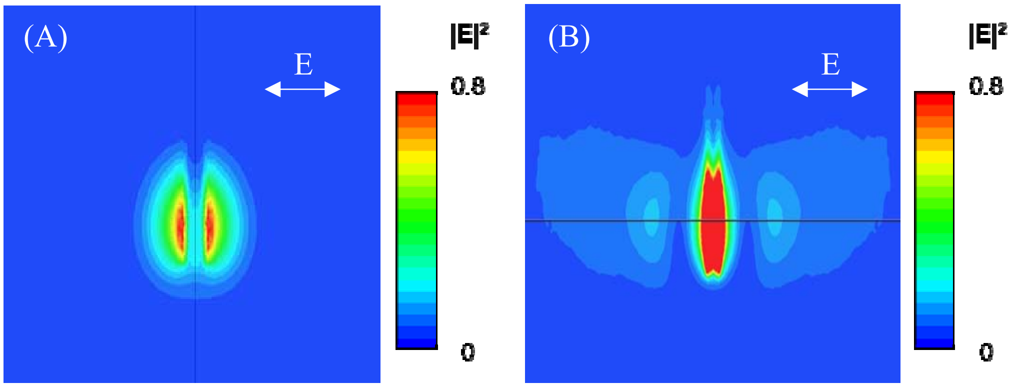

Figure S3. Intensity distribution at the surface of the photoresist for A) laser fluence of 0.01 J/cm^2 per pulse and B) laser fluence of 0.02 J/cm^2 per pulse. The incident electric field is taken as 1 V/m in these simulations.

4 Calculation of temperature rise in SU-8

The electromagnetic field distribution and the absorption profile obtained from the electromagnetic simulation were then input into a thermal simulation model using a similar FEM based solver from ANSYS. The absorption profile beneath the SU-8 surface is shown in Figure S4A and Figure S4B corresponding to the laser fluence of 0.01 J/cm^2 and 0.02 J/cm^2, respectively. In Figure S5B, the absorption at the center groove is smaller than that at the side

lobes due to the evaporation of SU-8 at the center by the laser, except at the tip of the groove which is high and lead to evaporation there. The energy absorbed within the volume of the photoresist was considered as a heat source in the thermal model which varied spatially according to the calculated absorption profile. Convection boundary conditions were assumed on the outer faces of the model with a convective heat transfer coefficient of 10W/m.K, and the accuracy of this value is not critical as the convection loss at the surface is orders of magnitude less than the input heat flux from the laser pulse. The temperature rise reaches its peak value at the end of the laser pulse. Figure S5A and Figure S5B show the peak temperature profile for incident laser fluence of 0.01 J/cm^2 and 0.02 J/cm^2, respectively. It is found that in these two cases, the maximum temperature rise in the central groove is 350°C and 280°C, higher than he boiling point of SU-8 of 130°C.[8] Furthermore, in the case of 0.02 J/cm^2 incident laser fluence, the hotspots at the two sides reach a temperature of 140°C, also exceeding the boiling point of SU-8, causing the evaporation of the photoresist and finally forming triple nanogrooves.

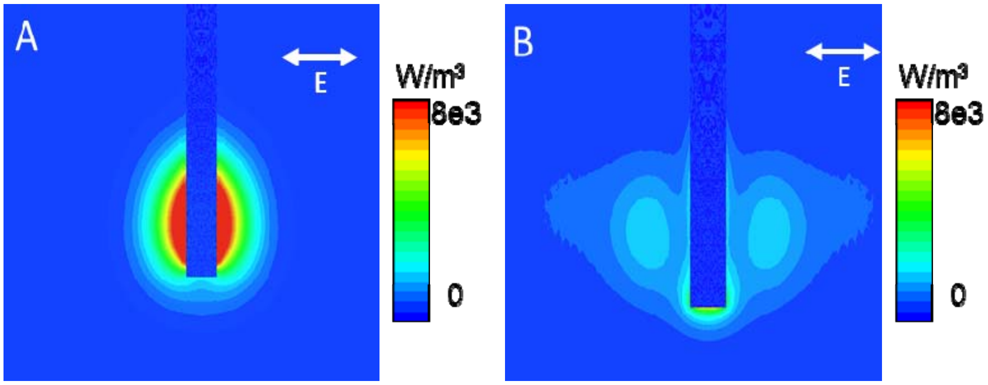

Figure S4. Absorption profile beneath the surface of the photoresist for A) laser fluence of 0.01 J/cm^2 per pulse and B) laser fluence of 0.02 J/cm^2 per pulse.

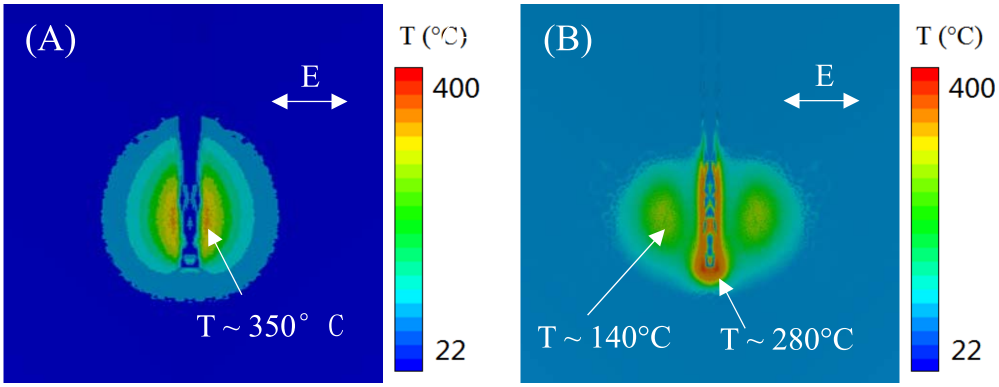

Figure S5. Temperature distribution of the surface of the photoresist at the end of a single laser pulse for A) laser fluence of 0.01 J/cm^2 per pulse and B) laser fluence of 0.02 J/cm^2 per pulse.

References

1. Webb G. W., Minin I. V. & Minin O.V. Variable Reference Phase in Diffractive Antennas: Review, Applications, New Results. IEEE Antenn. Propag. M. **53**, 77-94 (2011).
2. Chao W., Kim J., Rekawa S., Fischer P. & Anderson E. H. Demonstration of 12 nm Resolution Fresnel Zone Plate Lens based Soft X-ray Microscopy. Opt. Exp. **17**, 17669-17677 (2009).
3. Cao Q. & Jahns J. Comprehensive focusing analysis of various Fresnel zone plates. J. Opt. Soc. Am. A **21**, 561-571 (2004).
4. Wu Q., Chowdhury I. H. & Xu X. Femtosecond laser absorption in fused silica: Numerical and experimental investigation. Phys. Rev. B **72**, 085128 (2005).
5. Campo A. D. & Greiner C. SU-8: a photoresist for high-aspect-ratio and 3D submicron lithography. J. Micromech. Microeng. **17**, 81–95 (2007).
6. Parida O. P. & Bhat N. Characterization of optical properties of SU-8 and fabrication of optical components. Int. Conf. Opt. and Photon (CSIO), Chandigarh, India. 2009, 1-4.
7. Prasad P. N., Mark J. E. & Fai T. J. Polymers and other advanced materials: emerging technologies and business opportunities. Springer Science and Business Media: New York, 1995, 523-528.
8. Material Safety Data Sheet. MICRO CHEM, [PDF on Internet]. Available from: https://louisville.edu/micronano/files/documents/safety-data-sheets-sds/SU82000.pdf.
